# Simulating Surgical Skills in Animals: Systematic Review, Costs & Acceptance Analyses

**DOI:** 10.3389/fvets.2020.570852

**Published:** 2020-09-30

**Authors:** Konstantin D. Bergmeister, Martin Aman, Anne Kramer, Thilo L. Schenck, Otto Riedl, Simeon C. Daeschler, Oskar C. Aszmann, Helga Bergmeister, Mohammad Golriz, Arianeb Mehrabi, Gabriel Hundeshagen, Perenlei Enkhbaatar, Michael P. Kinsky, Bruno K. Podesser

**Affiliations:** ^1^Clinical Laboratory for Bionic Extremity Reconstruction, Department of Surgery, Medical University of Vienna, Vienna, Austria; ^2^Ludwig Boltzmann Institute for Cardiovascular Research at the Center for Biomedical Research, Medical University of Vienna, Vienna, Austria; ^3^Karl Landsteiner University of Health Sciences, Department of Plastic, Aesthetic and Reconstructive Surgery, University Hospital St. Pölten, Krems, Austria; ^4^Center for Biomedical Research, Medical University of Vienna, Vienna, Austria; ^5^Hand Surgery, Plastic Surgery and Aesthetic Surgery, Ludwig-Maximilians-University, Munich, Germany; ^6^Department of Hand, Plastic and Reconstructive Surgery, Burn Center, BG Trauma Hospital Ludwigshafen, Department of Plastic and Hand Surgery, University of Heidelberg, Ludwigshafen, Germany; ^7^Department of General, Visceral and Transplantation Surgery, University Hospital of Heidelberg, Heidelberg, Germany; ^8^Department of Anesthesiology, The University of Texas Medical Branch, Galveston, TX, United States

**Keywords:** surgical education, survey, cost analysis, surgical training, animal model

## Abstract

**Background:** Modern surgery demands high-quality and reproducibility. Due to new working directives, resident duty hours have been restricted and evidence exists that pure on-the-job training provides insufficient exposure. We hypothesize that supplemental simulations in animal models provide a realistic training to augment clinical experiences. This study reviews surgical training models, their costs and survey results illustrating academic acceptance.

**Methods:** Animal models were identified by literature research. Costs were analyzed from multiple German and Austrian training programs. A survey on their acceptance was conducted among faculty and medical students.

**Results:** 915 articles were analyzed, thereof 91 studies described *in-vivo* animal training models, predominantly for laparoscopy (30%) and microsurgery (24%). Cost-analysis revealed single-training costs between 307€ and 5,861€ depending on model and discipline. Survey results illustrated that 69% of the participants had no experience, but 66% would attend training under experienced supervision. Perceived public acceptance was rated intermediate by medical staff and students (4.26; 1–low, 10 high).

**Conclusion:** Training in animals is well-established and was rated worth attending in a majority of a representative cohort to acquire key surgical skills, in light of reduced clinical exposure. Animal models may therefore supplement the training of tomorrow's surgeons to overcome limited hands-on experience until virtual simulations can provide such educational tools.

## Background

Reproducibility of key surgical techniques with high-quality is of uttermost importance to ensure patient well-being, prevent secondary morbidity and its consequences. Essential surgical skills have traditionally been taught on-the-job, where intensive resident programs provided sufficient clinical exposure. However, due to the restricted working hours enforced for residents, these traditional on-the-job training paradigms could be insufficient to learn key surgical skills and thus alternative approaches required (1). For this purpose, supplemental education of key surgical techniques in animal models may provide a safe, realistic, and instructive training to augment clinical experiences and enhance patient safety.

The concept of surgical simulation has been introduced to medical schools to simulate vital clinical situations in a structured and realistic environment (2, 3). Here, simple clinical scenarios are simulated to train the key components of a specific basic skill, as for example suturing exercises on skin-like plastic devices. However, more complex clinical situations may require an *in-vivo* environment such as animal training to safely learn essential surgical skills. *In-vivo* skill-training can range from very standardized procedures such as microsurgical vessel anastomosis to complex simulations involving transplantation, intestine surgery, intraabdominal bleeding complications or endoscopic surgery training (4–8). Furthermore, *in-vivo* team trainings have been invented that include all operating room (OR) staff to simulate a specific situation such as for example intraabdominal bleeding in a large animal, to optimize teamwork in critical situations.

Despite the need to augment clinical training and ensure reproducibility of surgical key techniques, no study has reviewed global application as well as investigated training costs and acceptance among staff of surgical training in animal models. This study aims at providing an overview of existing animal models for surgical training and a cost-analysis of various training applications. Additionally, we discuss ethical issues associated with using animal models for surgical training and survey results illustrating faculty and students' opinions toward training in animal models.

## Methods

### Systematic Literature Review

As recommended by the PRISMA guidelines, the authors designed a systematic search strategy for PubMed, Google Scholar, Web of Science core collection and Scopus, to identify all potentially relevant studies for this review (see Supplementary Table 1). Search terms and exclusion criteria are listed in the Supplementary Text S2, 3. Included were all studies describing animal models for surgical training or assessing their suitability for the relevant approach. The date of the last search for each database was March 31st, 2020. The results of this systematic search were screened for possible inclusion against a predetermined checklist of inclusion criteria (Table 1). All publications using animal models for surgical training purposes in German or English were included. Two levels of screenings were used for 915 citations. First, titles and abstracts were screened to identify all potentially eligible studies. Studies meeting the inclusion criteria were obtained in full text and assessed thoroughly for eligibility. Studies not meeting the inclusion criteria were excluded. Additional information about the detailed search strategies is located in the supplemental material (Supplementary Text S3). The reference lists of the included literature were used to identify further relevant publications. Three reviewers independently applied the criteria for inclusion in reviewing the retrieved articles. If any differences were perceived toward inclusion, they were resolved by discussion among the reviewers. All relevant data including animal model, medical specialty, simulated intervention, year and origin were extracted (Table 1) and the included studies were evaluated for methodological quality, guided by the Cochrane Handbook for Systematic Reviews of Interventions. The risk of bias and manuscript quality control was assessed using the Cochrane risk of bias tool (Version 2).

**Table 1:** Inclusion criteria.

**Inclusion criteria**	
Population	*In-vivo* animal models
Intervention	Surgical training
Comparison	Alternative surgical trainings
	None
Outcome /Analyzed parameters	Date and Origin of publication
	Medical specialty
	Simulated intervention
	Animal model
Study characteristics	Peer-reviewed
	Date of publication 2000–2020
	German or English language
	Full publication accessible

*Based on the PICOS aspects (participants, interventions, comparisons, outcomes, and study characteristics) this table shows the inclusion criteria for considering studies for this systematic review*.

### Cost Analyses

Several medical training programs in academic institutions across Austria and Germany were analyzed for the costs of conducting experimental surgical training models (Table 2). At the Medical University of Vienna, we analyzed two large animal model trainings, a cardiac surgery team training and a training for management of surgical bleeding. Also, three different small animal training courses for microvascular anastomosis were analyzed from the Medical Universities of Vienna and Munich. Additionally, we analyzed a 1-day workshop for general and visceral surgery at the University Hospital of Heidelberg. For each training session, costs for animal acquisitions and husbandry, perioperative care and material as well as staff and institutional costs were analyzed, and total cost per training were calculated. All cost analyses are based on non-profit in-house costs without value-added tax and without costs for teaching staff. All trainings were performed in compliance with the principles of laboratory animal care as recommended by FELASA (9). Approval was obtained from the local ethics committees, for Austria being the Medical University of Vienna and the Austrian Ministry for Research and Science and for Germany being the University of Heidelberg (4) or the University of Munich (both in accordance with the German animal welfare act).

**Table 2:** Cost analyses of various surgical trainings in animal models.

**Item**	**Costs**			
**Training model:**	**1. Cardiac surgery—team training**	**2. Microsurgery**	**3. Abdominal bleeding—team training**	**4. Transplant surgery**
Animal (acquisition, transport, facilities)	634	50	334	320
Anesthesia and perioperative care (equipment, staff, medication, heart lung machine)	2,581	66	481	350
Material (sutures, specific devices, single use equipment)	1,344	76	119	500
Institutional costs (administration, finances, operating room)	1,300	115	1,300	920
Total	5,861€	307€	2,234€	2,090€

***1:** Cardiac surgery training costs: Table of the costs that resulted from one training event (one day) for open heart surgery on a single pig. Total costs were 5,861€ (6,574 USD) that resulted from expenses for material, animal-related costs, anesthesia, and institutional costs. **2:** Microsurgery training costs: Shown are the costs that resulted from one training event (one day) for microsurgery on a single rat. Total costs were 307€ (344 USD) that resulted from expenses for material, animal-related costs, anesthesia, and institutional costs. **3:** Abdominal bleeding team training costs: Displayed are the costs that resulted from one training event (one day) for four trainees on a single pig. Total costs were 2,234€ (2,506 USD) that resulted from expenses for material, animal-related costs, anesthesia, and institutional costs. **4:** Transplantation surgery team-training costs: Shown are the costs that resulted from one training event (1 day) for four trainees on a single pig. Total costs were 2,090€ (2,344 USD) that resulted from expenses for material, animal-related costs, anesthesia, and institutional costs*.

### Survey on Acceptance of Animal Models

In an in-house survey at the Medical University of Vienna, we investigated the opinions toward training in animal models of 10,335 M.D. and Ph.D. students and 3,824 staff members. The survey was conducted using the MedCampus computer system (CAMPUSOnline Graz, Austria) of the Medical University of Vienna, which is accessible to all students and staff members. The survey was prepared from January to October 2015 and conducted during November 2015. Statistics were conducted using SPSS (V.21, IBM Corp, US). For the survey, approval was obtained from the Medical University of Vienna's data privacy committee. The in-house survey was performed in accordance with all relevant guidelines and regulations.

## Results

### Systematic Literature Review

**Figure 1:**
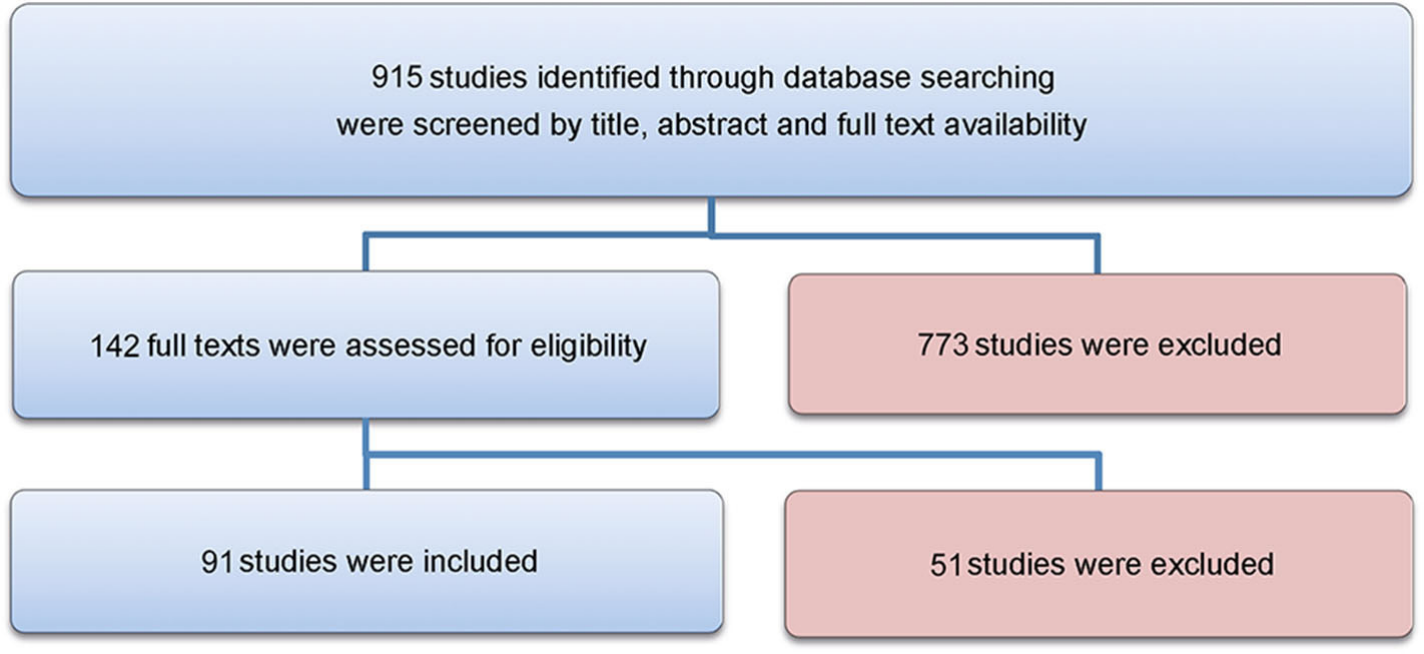
Flowchart. The study selection process is visualized in this Figure, as recommended by the PRISMA guidelines.

**Figure 2:**
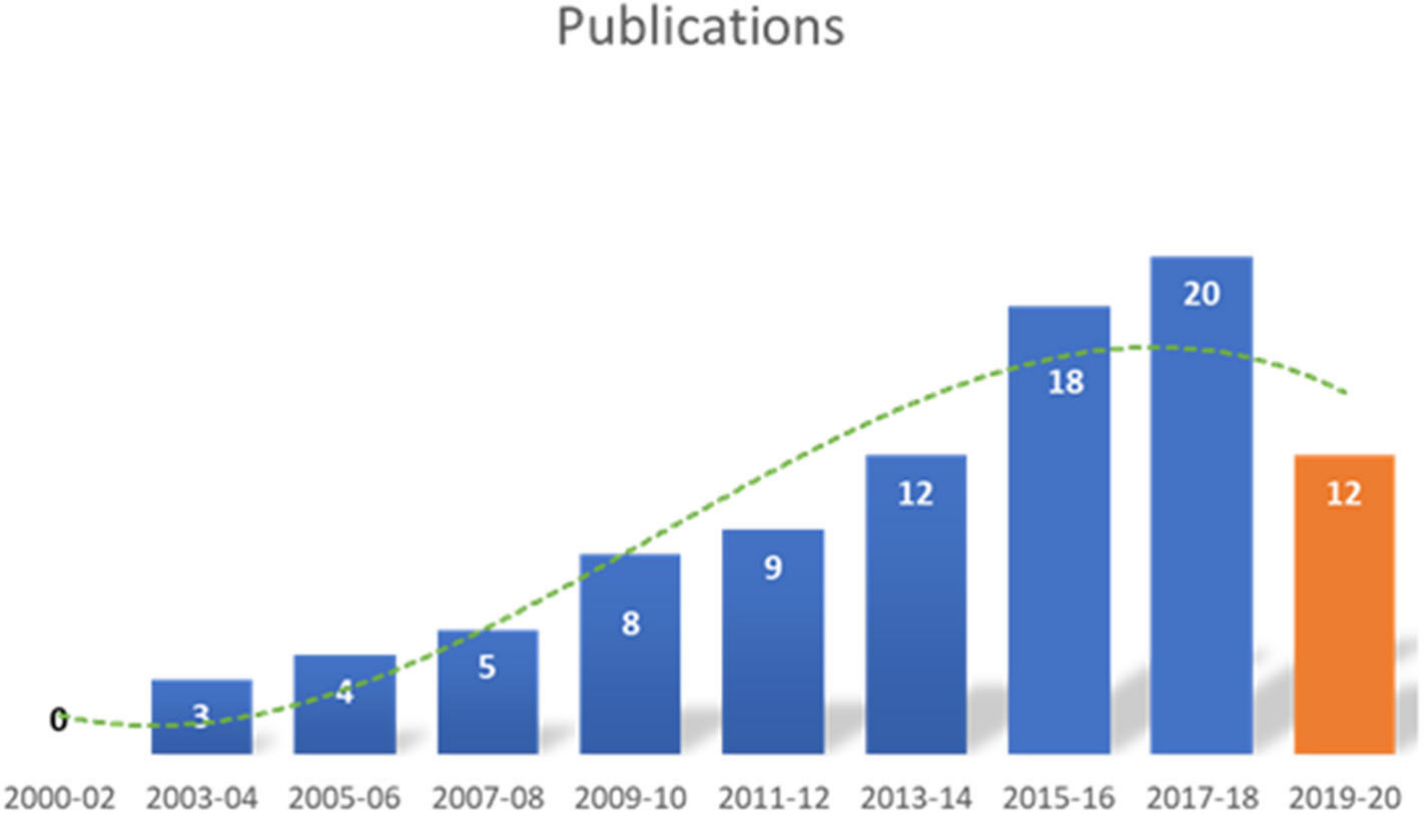
Date of included publications. The number of publications identified in this analysis, increased from 2000 to 2020. The decline in 2019–2020 is most likely due to only including the first three months of 2020.

**Figure 3:**
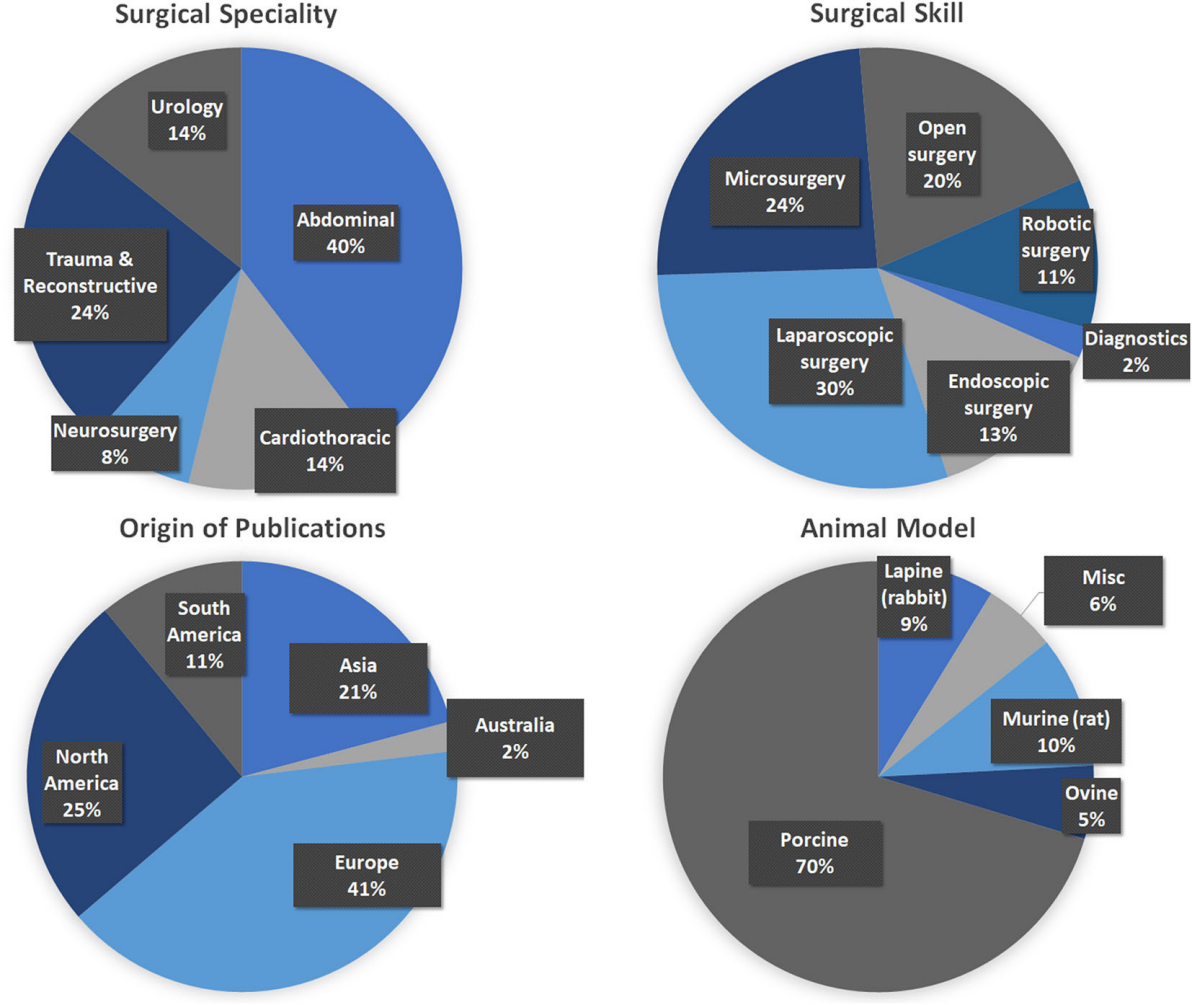
Analyses of the included publications. **(Top Left)** Surgical specialty that was simulated. **(Top Right)** Surgical skill that was trained (Please note that for reasons of clarification, laparoscopic surgery was not included into endoscopic surgery.) **(Bottom Left)** The origin of the included publications was identified by first and last authorship. **(Bottom Right)** The type of animal models used for simulation.

A total of 915 articles matched the search terms and 142 were included in further analyses following title and abstract screening. Thereof, 51 full-text studies were excluded from further analyses based on the exclusion criteria (Supplementary Text 2). A total of 91 studies were included in the analyses and analyzed for further information (Supplementary Table 1). Figure 1 illustrates the study selection process. All 91 publications included in the analysis were screened using the Cochrane risk of bias analysis tool (Version 2) and judged for their methodological quality and risk of bias, guided by the Cochrane Handbook for Systematic Reviews of Interventions. Overall the studies included all relevant information on the proposed surgical training models in animals. In this regard, no risk of bias was found in terms of selection bias, performance bias, detection bias, attrition bias, reporting bias, or other bias. Therefore, the risk of bias was rated by both the automated algorithm and the evaluator as low and the overall quality of the included studies rated as standard quality. Analyses of the date of publication revealed a constant increase of publications from 2000 to 2018. For 2019-2020, the sum is lower due to only 3 months included for 2020 (Figure 2). The origin of the publication was determined by first and last authorship, indicating that the majority of publications were from Europe (41%), followed by North America (25%), Asia (21%) and South America (11%) (Figure 3). Most publications described applications in abdominal surgery (40%), trauma & reconstructive surgery (24%), urology (14%), and cardiothoracic surgery (14%). The most frequent simulation was laparoscopic surgery (30%) and microsurgery (24%), (Figure 3). The majority of simulations were conducted in porcine models (70%), with a clear trend toward large animal models (75%) (Figure 3).

### Cost Analyses

#### Large Animal -Team Training: Cardiac Surgery:

Six cardiac surgeons (resident and consultant) trained open-heart procedures (valve replacement surgery) on a single pig. For this purpose, the animal was adequately anesthetized and continuously monitored throughout the training unit, lasting ~6 h. A veterinarian and a cardio technician were responsible for anesthesia and extracorporal circulation. Reported are all costs that resulted from the training, animal purchase and husbandry, medication, surgical and medical supplies including heart-lung machine (HLM) equipment and institutional costs. Overall costs were 5,861€ (6,574 USD) for one simulation. Reported are mean values of three single day events. (Table 2).

#### Single Person Training: Microsurgery

Microsurgery training models were analyzed from three different institutions in Germany and Austria. Single trainees (resident or consultant) practiced microsurgical anastomoses and nerve coaptation using a rat model. For this purpose, the animal was placed on mechanical ventilator and continuously monitored throughout the training unit, which lasted ~4 h. A veterinarian was present at all times to control appropriate anesthesia and analgesia. Training was performed using a regular surgical microscope. Overall costs were 307€ (344 USD) for one simulation per trainee. Reported are means of three different training programs, calculated to present the costs for a single trainee (Table 2).

#### Team Training: Management of Surgical Bleeding Complications

Groups of four trainees of resident level trained acute abdominal bleeding situations in a porcine model supervised by one instructor in. Two training stations included a total of eight trainees practicing different bleeding scenarios and locations. For this purpose, the animal was anesthetized and continuously monitored throughout the training unit, which lasted a total of 4 h. Continuous warming of the animal and compensation of blood loss was essential to prevent circulatory complications. A veterinarian was responsible for anesthesia during surgery and terminal euthanasia after completion of the training unit. The costs were calculated per four trainees on one animal for ~4 h. (Table 2).

####  Team Training: Transplantation Surgery Training

Groups of three to four trainees of resident or junior consultant level were supervised by one senior consultant (general and visceral surgery) with sufficient experience in a large porcine model. 12 to 16 trainees practiced for up to 8 h on four different training stations to practice various surgical techniques and scenarios with special regard to organ transplantation. For this purpose, the animal was generally anesthetized and continuously monitored throughout the training unit. Continuous warming of the animal and compensation of blood loss was essential to prevent circulatory complications. An experienced consultant was responsible for anesthesia during surgery and terminal euthanasia after completion of the training unit. The costs were calculated per four trainees on one animal for ~8 h and staff costs are not included (Table 2).

### Survey on Acceptance of Animal Models

**Figure 4:**
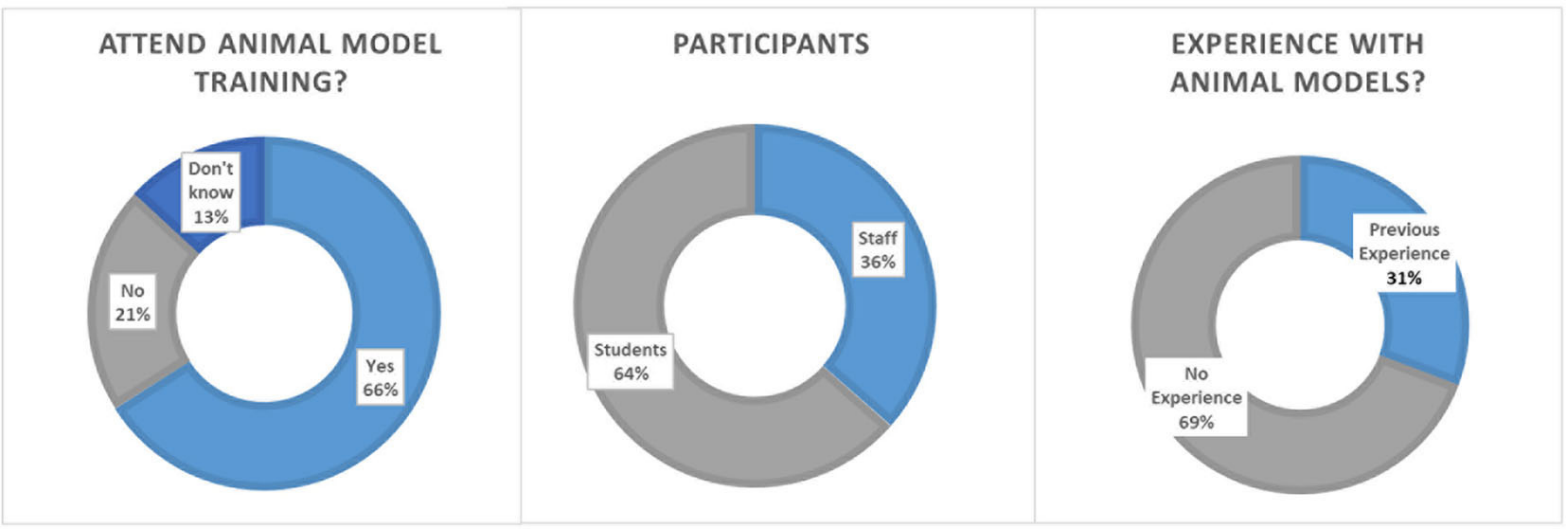
Survey results on academic acceptance toward animal models in surgical training. **(Left)** Details of the participants. **(Middle)** Number of participants with experience with animal models. **(Right)** Opinions toward participating an animal training model for surgery.

Overall, 906 participants completed the in-house online survey. Participants were 36.5% staff members (medical or research staff) and 63.5% medical or Ph.D. students. Participants rated the importance of continuous training in the job at 9.08 ± 2.05 (scale 1–10; 1 not important and 10 most important) and team trainings likewise high at 8.92 ± 1.50 (scale 1–10; 1 not important and 10 most important, 8.1% voted “don't know”). A majority of 69.24% did not have any prior experience with animal models for training purposes. Whether participants could imagine using animal models for medical training purposes was rated at 7.02 ± 2.66 (1–absolutely not, 10 very much; 10% voted “don't know”). When asked if they would attend a surgical training on an animal model under the supervision of experienced staff 65.94% responded yes, 20.91% no and 13.14% voted “don't know.” The perceived public acceptance of animal models for training purposes was rated low to intermediate at 4.26 ± 1.77 by medical staff and students (1–not accepted, 10 very much accepted; Figure 4).

## Discussion

First analyses indicate that the restricted exposure may negatively impact patient outcomes and surgical performance (10, 11) and thus alternative methods are required to teach key surgical skills. This is further aggravated by the steep learning curves of many newly developed technologies (e.g., robotic or supermicro-surgery), limited resources in times of high-cost pressure, more complex cases and generally sicker patients (3). Additionally, the overall number of surgeries both in the US and in Europe have increased in the past decade, particularly highly specialized procedures such as laparoscopy or robotic surgery (12, 13). The use of animal models to simulate realistic surgical scenarios may compensate these developments but has not been systematically explored.

Our results show that the number of publications regarding *in-vivo* animal models for surgical training has constantly increased from 2000 to 2018. The majority of these publications origin from Europe (41%), where in 2003 the European Union limited the maximum working hours for residents to 56 h and in 2009 to 48 hours per week [European Directive 2003/88/EG (14)]. Similar trends are seen in North America, where in 2003 the Accreditation Council for Graduate Medical Education (ACGME) introduced national work hour regulations, which were further restricted in 2011 (10, 15, 16). Therefore, we assume that surgeons use these models to compensate for lost training opportunities in a safe environment, yet no statistics exist on their use. However, exemplary studies have shown their efficiency to improve learning of key surgical skills (17–21).

The most commonly simulated technique in these publications was the use of endoscopic and laparoscopic surgery, which combined for a total of 43%. Including robotic surgery and microsurgery, over 75% of the analyzed publications simulated specific technical skills with modern technical devices. This majority likely results from the steep learning curve of these particular skills before the procedure can be safely applied in patients (22, 23). The goal of these simulations is to enhance surgical reproducibility, which refers to being able to achieve a target outcome with sufficient quality with a certain minimum probability. In practice, this translates to e.g., sufficiently replacing a cardiac valve with high long-term survival or reconstructing a defect by free flap surgery without flap failure in at least 95% (24). In both scenarios and any other alike surgical key components, such as microsurgery anastomosis of vessels or valve fitting, define the success of these surgeries. While some publications suggest that these key technical elements as for example microsurgery can also be taught in *ex-vivo* models, many studies have shown that trainees highly appreciate realistic living models with realistic tissue feel, perfusion, and anatomical representation, before translating these skills to patients (6, 6, 8, 25, 26). Furthermore, these models enable surgeons to perform a specific operation multiple times within a short time period thereby improving skill acquisition. For example, an analysis of a transplantation hands-on course in a porcine model at the University Hospital of Heidelberg showed that each participant could perform on average, 1.8 multiorgan procurements, 2.3 kidney, 1.5 liver, and 0.7 pancreas transplantations within just 2 days (4). In comparison, clinical exposure for highly-specialiced surgeries such as transplantation surgery, free flap surgery or robotic surgery may be limited to few per year. In our analyses, most publications originated from surgical specialties, where surgical failure may result in high patient morbidity or ultimately death, and thus possibly compromise patient safety.

To create realistic, non-patient environment, large animal models are used for skills training in cardiac and abdominal surgery, which allowed accurate simulation as well as simultaneous education of several surgeons. However, the costs for large animal models are substantial, even in a non-profit university setting as described above, where many routine costs are covered by the institution. The actual training costs are therefore likely higher, if a fully equipped operating room for biomedical research and training is not available. The costs analyses are limited to Austria and Germany and may vary for other countries or institutions. Yet, they provide accurate information regarding the multiple origins of training costs that have to be considered (27). Surprisingly, small animal models were underrepresented in our analyses, possibly because of the many long-established models, and subsequent few publications in the past years.

Because animal models for training are an ethically difficult subject, the acceptance of such models within an institution is a major aspect of its establishment and success. At our institutions with a history of animal models for surgical training, 30% of the surveys' participants said to have been exposed to these simulations. Potentially as a consequence, the acceptance of these models was high and 66% would participate under the supervision of experienced staff. The acceptance rate among staff after successful completion of animal training might be even higher, indicated by Daly et al. (27), who found a rate of over 90% of acceptation and recommendation rate in medical students after performing surgery in animal models (27). Likewise, a recent survey in 3,096 members of the public indicates that 55% agreed that training in animals is necessary at medical universities, while 33% did not know and only 12% did not believe it to be a necessity (28). Yet in our survey, participants rated the public acceptance of these training models only moderately, which may misjudge public support for proper training in animal models. This aspect may be particularly relevant for finding funding for these simulations, especially in countries where *in-vivo* research is publicly less accepted (29). Currently, animal right activism groups are very active in the US and Europe and oppose animal experiments and supposedly also training in live animals with large scale lobbying, as for example the 2015 “Stop Vivi-section” initiative (30). We agree, that the use of animals for biomedical research and surgical training is ethically difficult (30) and must be done with the highest regards to its ethical implications and performed according to national standards. Also, we believe it may currently be a necessity to compensate for the decline in training opportunities during residency for high-risk surgeries in a safe environment. Therefore, its impact on providing a substantial benefit to our health systems is significant, while the increase in relation to the global number of research animals would be comparably small (31). The concept of 3Rs, must however always be applied for animal models in surgical training in order to reduce their use, replace *in-vivo* with *ex-vivo* models whenever possible and refine models to maximize educational benefit. Ultimately, animal models for surgical training might be sufficiently replaced by realistic simulations. At the moment, many reviews demonstrate that medical simulations currently lack technical quality and effectiveness to produce reliable results (32, 33). Valid models comparing simulation to clinical care continues to be a question that has been unanswered (34, 35). Contrary, animal models, especially large animals, while not inexpensive, provide unique realistic physiologic challenges that often cannot be captured by simulation modules. The learners will be able to sense true host physiologic variations as well as pathological responses that occur in this complex *in-vivo* environment, that has currently not been matched by simulations.

## Conclusion

Our study shows that many surgical simulations in animal models have been described and are well-accepted among staff at an exemplary institution as indicated by a representative survey of academic students and staff to compensate for inadequate clinical exposure. Following the 3R principle, a concept of merging locally available experimental models and surgical *in-vivo* training represents a reasonable approach to maximize cost efficiency and general acceptance. Yet, these models should not be considered as an alternative to hands-on training in patients but a supplemental tool to learn key surgical skills (7). In the future, modern virtual reality simulations may hopefully provide such training models. This would allow training without the ethically disputable compromise of using animals.

## Data Availability Statement

The datasets used and/or analysed during the current study are either available in the supplemental file or from the corresponding author on reasonable request.

## Author Contributions

KB, MA, and BP designed the concept. KB, MA, AK, TS, OR, SD, OA, HB, MG, AM, GH, PE, MK, and BP analyzed data, contributed their specific expertise, wrote the manuscript, and revised it critically. All authors have read and approved the manuscript.

## Conflict of Interest

The authors declare that the research was conducted in the absence of any commercial or financial relationships that could be construed as a potential conflict of interest.

## Abbreviations

ACGME: Accreditation Council for Graduate Medical Education
FELASA: Federation of European Laboratory Animal Science Associations
M.D.: Medical Doctor
Ph.D.: Doctor of Philosophy
HLM: heart lung machine.
