# Supplementary material for: Simulating Surgical Skills in Animals: Systematic Review, Costs & Acceptance Analyses
Source: Front Vet Sci. 2020 Sep 30;7:570852. doi: 10.3389/fvets.2020.570852 (PMC7554573; doi:10.3389/fvets.2020.570852)
Supplement: Supplementary file 1 [file Table_1.DOCX]

**Supplemental Material**

**S1 Table: Meta-Analysis:**

List of all papers included in the analysis including details on origin, year of publication and trained skill.

| Publication | Year | Speciality | Skill | Continent | Animal |
| --- | --- | --- | --- | --- | --- |
| Araujo, S. E., et al. (2014). “Short-duration virtual reality simulation training positively impacts performance during laparoscopic colectomy in animal model: results of a single-blinded randomized trial : VR warm-up for laparoscopic colectomy.” Surg Endosc 28(9): 2547-2554. | 2014 | Abdominal | Laparoscopic surgery | South America | porcine |
| Camus, M., et al. (2013). “Validation of a live animal model for training in endoscopic hemostasis of upper gastrointestinal bleeding ulcers.” Endoscopy 45(6): 451-457. | 2013 | Abdominal | Endoscopic surgery | Europe | porcine |
| Cruz, J. A., et al. (2012). “Surgical performance during laparoscopic radical nephrectomy is improved with training in a porcine model.” J Endourol 26(3): 278-282. | 2012 | Abdominal | Laparoscopic surgery | South America | lapine (rabbit) |
| de la Torre, M., et al. (2014). “Uniportal video-assisted thoracoscopic lobectomy in the animal model.” J Thorac Dis 6(Suppl 6): S656-659. | 2014 | Cardiothoracic | Endoscopic surgery | Europe | ovine |
| Diaz-Guemes Martin-Portugues, I., et al. (2013). “Ureteral obstruction swine model through laparoscopy and single port for training on laparoscopic pyeloplasty.” Int J Med Sci 10(8): 1047-1052. | 2013 | Urology | Laparoscopic surgery | Europe | porcine |
| Enciso, S., et al. (2016). “Validation of a model of intensive training in digestive laparoscopic surgery.” Cir Esp 94(2): 70-76. | 2016 | Abdominal | Laparoscopic surgery | Europe | porcine |
| Esposito, C., et al. (2016). “Training Models in Pediatric Minimally Invasive Surgery: Rabbit Model Versus Porcine Model: A Comparative Study.” J Laparoendosc Adv Surg Tech A 26(1): 79-84. | 2016 | Abdominal | Laparoscopic surgery | Europe | porcine |
| Fearn, S. J., et al. (2006). “A laparoscopic access technique for endovascular procedures: surgeon training in an animal model.” J Endovasc Ther 13(3): 350-356. | 2006 | Abdominal | Laparoscopic surgery | Australia | porcine |
| Fernandez-Miranda, J. C., et al. (2010). “Animal model for endoscopic neurosurgical training: technical note.” Minim Invasive Neurosurg 53(5-6): 286-289. | 2010 | Neurosurgery | Endoscopic surgery | North America | murine (rat) |
| Foletti, J. M., et al. (2013). “Endoscopic treatment of mandibular condylar fractures in live minipigs: benefits of the operative learning curve.” Br J Oral Maxillofac Surg 51(7): 630-633. | 2013 | Trauma & Reconstructive | Endoscopic surgery | Europe | porcine |
| Fu, B., et al. (2007). “New model for training in laparoscopic dismembered ureteropyeloplasty.” J Endourol 21(11): 1381-1385. | 2007 | Urology | Laparoscopic surgery | Asia | porcine |
| Hall, A. B. (2011). “Randomized objective comparison of live tissue training versus simulators for emergency procedures.” Am Surg 77(5): 561-565. | 2011 | Cardiothoracic | Open surgery | North America | porcine |
| Heinrich, M., et al. (2006). “Comparison of different training models for laparoscopic surgery in neonates and small infants.” Surg Endosc 20(4): 641-644. | 2006 | Abdominal | Laparoscopic surgery | Europe | lapine (rabbit) |
| Hernandez Mondragon, O. V., et al. (2015). “The Per Oral Endoscopic Myotomy (POEM) technique: how many preclinical procedures are needed to master it?” Endosc Int Open 3(6): E559-565. | 2015 | Abdominal | Endoscopic surgery | North America | porcine |
| Izawa, Y., et al. (2016). “Ex-vivo and live animal models are equally effective training for the management of a penetrating cardiac injury.” World J Emerg Surg 11(1): 45. | 2016 | Cardiothoracic | Open surgery | Asia | porcine |
| Kajiwara, N., et al. (2011). “Training in robotic surgery using the da Vinci® surgical system for left pneumonectomy and lymph node dissection in an animal model.” Ann Thorac Cardiovasc Surg 17(5): 446-453. | 2011 | Cardiothoracic | Robotic surgery | Asia | porcine |
| Kirlum, H. J., et al. (2005). “Advanced paediatric laparoscopic surgery: repetitive training in a rabbit model provides superior skills for live operations.” Eur J Pediatr Surg 15(3): 149-152. | 2005 | Abdominal | Laparoscopic surgery | Europe | lapine (rabbit) |
| La Torre, M. and C. Caruso (2012). “Resident training in laparoscopic colorectal surgery: role of the porcine model.” World J Surg 36(9): 2015-2020. | 2012 | Abdominal | Laparoscopic surgery | Europe | porcine |
| Leclere, F. M., et al. (2013). “Microsurgery and liver research: Lumbricus terrestris, a reliable animal model for training?” Clin Res Hepatol Gastroenterol 37(2): 166-170. | 2013 | Abdominal | Microsurgery | Europe | misc |
| Leclere, F. M., et al. (2013). “Is there good simulation basic training for end-to-side vascular microanastomoses?” Aesthetic Plast Surg 37(2): 454-458. | 2013 | Trauma & Reconstructive | Microsurgery | Europe | misc |
| Liu, X., et al. (2016). “A Secure and High-Fidelity Live Animal Model for Off-Pump Coronary Bypass Surgery Training.” J Surg Educ 73(4): 583-588. | 2016 | Cardiothoracic | Microsurgery | Asia | porcine |
| Marchini, G. S., et al. (2016). “Specific training for LESS surgery results from a prospective study in the animal model.” Int Braz J Urol 42(1): 90-95. | 2016 | Urology | Laparoscopic surgery | South America | porcine |
| Mehrabi, A., et al. (2006). “Development and evaluation of a training module for the clinical introduction of the da Vinci robotic system in visceral and vascular surgery.” Surg Endosc 20(9): 1376-1382. | 2006 | Abdominal | Robotic surgery | Europe | murine (rat) |
| Passerotti, C. C., et al. (2009). “Comparing the quality of the suture anastomosis and the learning curves associated with performing open, freehand, and robotic-assisted laparoscopic pyeloplasty in a swine animal model.” J Am Coll Surg 208(4): 576-586. | 2009 | Urology | Laparoscopic surgery | North America | porcine |
| Training microneurosurgery - four years experiences with an in vivo model. Regelsberger , Heese O, Horn P, Kirsch M, Eicker S, Sabel M, Westphal M. | 2011 | Neurosurgery | Microsurgery | Europe | porcine |
| Regelsberger, J., et al. (2015). “In vivo porcine training model for cranial neurosurgery.” Neurosurg Rev 38(1): 157-163; discussion 163. | 2015 | Neurosurgery | Microsurgery | Europe | porcine |
| Riccetto, C. L., et al. (2007). “Experimental animal model for training transobturator and retropubic sling techniques.” Urol Int 78(2): 130-134. | 2007 | Urology | Open surgery | South America | ovine |
| Rosenberg, J., et al. (2013). “An animal model to train Lichtenstein inguinal hernia repair.” Hernia 17(2): 255-258. | 2013 | Abdominal | Open surgery | Europe | porcine |
| Ryska, O., et al. (2016). “A new experimental model of calculous cholecystitis suitable for the evaluation and training of minimally invasive approaches to cholecystectomy.” Surg Endosc. | 2016 | Abdominal | Laparoscopic surgery | Europe | porcine |
| Schleimer, K., et al. (2012). “Training a sophisticated microsurgical technique: interposition of external jugular vein graft in the common carotid artery in rats.” J Vis Exp(69). | 2012 | Trauma & Reconstructive | Microsurgery | Europe | murine (rat) |
| Simforoosh, N., et al. (2011). “Laparoscopic animal surgery for training without sacrificing animals; introducing the rabbit as a model for infantile laparoscopy.” J Laparoendosc Adv Surg Tech A 21(10): 929-933. | 2011 | Abdominal | Laparoscopic surgery | Asia | lapine (rabbit) |
| Soria, F., et al. (2015). “Development and Validation of a Novel Skills Training Model for Retrograde Intrarenal Surgery.” J Endourol 29(11): 1276-1281. | 2015 | Urology | Endoscopic surgery | Europe | porcine |
| Spetzger, U., et al. (2011). “Training models for vascular microneurosurgery.” Acta Neurochir Suppl 112: 115-119. | 2011 | Neurosurgery | Microsurgery | Europe | lapine (rabbit) |
| Sundermann, S. H., et al. (2016). “Two- and three-dimensional transoesophageal echocardiography in large swine used as model for transcatheter heart valve therapies: standard planes and values.” Interact Cardiovasc Thorac Surg 22(5): 580-586. | 2016 | Cardiothoracic | Diagnostics | Europe | porcine |
| Tedde, M. L., et al. (2015). “Video-assisted thoracoscopic surgery in swine: an animal model for thoracoscopic lobectomy training.” Interact Cardiovasc Thorac Surg 21(2): 224-230. | 2015 | Cardiothoracic | Endoscopic surgery | South America | porcine |
| Teh, S. H., et al. (2007). “A suitable animal model for laparoscopic hepatic resection training.” Surg Endosc 21(10): 1738-1744. | 2007 | Abdominal | Laparoscopic surgery | North America | ovine |
| La Torre M^1^, Caruso C (2013).The animal model in advanced laparoscopy resident training.Surg Laparosc Endosc Percutan Tech. 2013 Jun;23(3):271-5. doi: 10.1097/SLE.0b013e31828b895b. | 2013 | Abdominal | Laparoscopic surgery | Europe | porcine |
| Park J^1^, Yim S, Eun SC. 2016 Experimental Design for Composite Face Transplantation J Craniofac Surg. 2016 Jun;27(4):843-5. | 2016 | Trauma & Reconstructive | Open surgery | Asia | porcine |
| Mouraviev V, Klein M, Schommer E, Thiel DD, Samavedi S, Kumar A, Leveillee RJ, Thomas R, Pow-Sang JM, Su LM, Mui E, Smith R, Patel V.Urology residents experience comparable workload profiles when performing live porcine nephrectomies and robotic surgery virtual reality training modules.J Robot Surg. 2016 Mar;10(1):49-56. doi: 10.1007/s11701-015-0540-1. Epub 2016 Jan 11. | 2016 | Urology | Robotic surgery | North America | porcine |
| Jaimovich SG, Bailez M, Asprea M, Jaimovich R. Neurosurgical training with simulators: a novel neuroendoscopy model.Childs Nerv Syst. 2016 Feb;32(2):345-9. doi: 10.1007/s00381-015-2936-7. Epub 2015 Oct 22. | 2015 | Neurosurgery | Endoscopic surgery | South america | murine (rat) |
| Rosenberg J, Presch I, Pommergaard HC, Burcharth J, Bagot d’Arc M.An animal model to train Lichtenstein inguinal hernia repair.Hernia. 2013 Apr;17(2):255-8. doi: 10.1007/s10029-012-0981-7. Epub 2012 Aug 21 | 2013 | Abdominal | Open surgery | europe | porcine |
| Baek RM, Eun SC, Heo CY, Chang H.Experimental facial transplantation surgery.J Craniofac Surg. 2010 May;21(3):648-51. doi: 10.1097/SCS.0b013e3181d84010. | 2010 | Trauma & Reconstructive | Microsurgery | Asia | lapine (rabbit) |
| Vázquez-Sequeiros E, de Miquel DB, Olcina JR, Martín JA, García M, Lucas DJ, Garrido E, González C, Blanco AP, Arnau MR, Buenadicha A, Vicente VM, de Argila CM, Milicua JM.Training model for teaching endoscopic submucosal dissection of gastric tumors.Rev Esp Enferm Dig. 2009 Aug;101(8):546-52. | 2009 | Abdominal | Laparoscopic surgery | Europe | porcine |
| Zhang X, Wang B, Ma X, Zhang G, Shi T, Ju Z, Wang C, Li H, Ai X, Fu B.Laparoscopic adrenalectomy for beginners without open counterpart experience: initial results under staged training.Urology. 2009 May;73(5):1061-5. doi: 10.1016/j.urology.2008.11.058. | 2009 | Urology | Laparoscopic surgery | Asia | porcine |
| Zhang X, Zhang GX, Wang BJ, Ma X, Fu B, Shi TP, Lang B, Wang C, Ju ZH, Ai X, Wu Z.A multimodal training program for laparoscopic pyeloplasty.J Endourol. 2009 Feb;23(2):307-11. doi: 10.1089/end.2008.0356. | 2009 | Urology | Laparoscopic surgery | Asia | porcine |
| Hall AB, Riojas R, Sharon D.Comparison of self-efficacy and its improvement after artificial simulator or live animal model emergency procedure training.Mil Med. 2014 Mar;179(3):320-3. doi: 10.7205/MILMED-D-12-00446. | 2014 | Trauma & Reconstructive | Open surgery | North America | porcine |
| Van Bruwaene S, Schijven MP, Napolitano D, De Win G, Miserez M Porcine cadaver organ or virtual-reality simulation training for laparoscopic cholecystectomy: a randomized, controlled trial.J Surg Educ. 2015 May-Jun;72(3):483-90. doi: 10.1016/j.jsurg.2014.11.015. Epub 2014 Dec 30. | 2015 | Abdominal | Laparoscopic surgery | Europe | porcine |
| Palter VN, Orzech N, Aggarwal R, Okrainec A, Grantcharov TP. Resident perceptions of advanced laparoscopic skills training.Surg Endosc. 2010 Nov;24(11):2830-4. doi: 10.1007/s00464-010-1058-2. Epub 2010 Apr 29. | 2010 | Abdominal | Laparoscopic surgery | North America | porcine |
| Phillips AB, Green J, Bergdall V, Yu J, Monreal G, Gerhardt M, Cheatham JP, Galantowicz M, Holzer RJ.Teaching the “hybrid approach”: a novel swine model of muscular ventricular septal defect.Pediatr Cardiol. 2009 Feb;30(2):114-8. doi: 10.1007/s00246-008-9297-x. Epub 2008 Aug 19. | 2009 | Cardiothoracic | Open surgery | North America | porcine |
| Chen VK, Marks JM, Wong RC, McGee MF, Faulx AL, Isenberg GA, Schomisc SJ, Deng CX, Ponsky JL, Chak A.Creation of an effective and reproducible nonsurvival porcine model that simulates actively bleeding peptic ulcers.Gastrointest Endosc. 2008 Sep;68(3):548-53. doi: 10.1016/j.gie.2008.03.1087. Epub 2008 Jul 11. | 2008 | Abdominal | Laparoscopic surgery | North America | porcine |
| Sterbis JR, Hanly EJ, Herman BC, Marohn MR, Broderick TJ, Shih SP, Harnett B, Doarn C, Schenkman NS.Transcontinental telesurgical nephrectomy using the da Vinci robot in a porcine model.Urology. 2008 May;71(5):971-3. doi: 10.1016/j.urology.2007.11.027. Epub 2008 Mar 4. | 2008 | Urology | Robotic surgery | North America | porcine |
| Rulli F, Cina G, Galatà G, Cina A, Vincenzoni C, Fiorentino A, Farinon AM.Teaching subfascial perforator veins surgery: survey on a 2-day hands-on course.ANZ J Surg. 2004 Dec;74(12):1116-9. | 2004 | Trauma & Reconstructive | Endoscopic surgery | Europe | porcine |
| Hanly EJ, Marohn MR, Bachman SL, Talamini MA, Hacker SO, Howard RS, Schenkman NS.Multiservice laparoscopic surgical training using the daVinci surgical system.Am J Surg. 2004 Feb;187(2):309-15. | 2004 | Abdominal | Robotic surgery | North America | porcine |
| Abu-Zidan, F. M., et al. (2004). “Establishment of a teaching animal model for sonographic diagnosis of trauma.” J Trauma 56(1): 99-104. | 2004 | Trauma & Reconstructive | Diagnostics | Asia | porcine |
| Alves, J. R., et al. (2012). “Animal model for training in sentinel lymph node biopsy of the stomach through combined methods.” Acta Cir Bras 27(12): 833-840. | 2012 | Abdominal | Open surgery | South America | lapine (rabbit) |
| Bodin, F., et al. (2015). “Porcine model for free-flap breast reconstruction training.” J Plast Reconstr Aesthet Surg 68(10): 1402-1409. | 2015 | Trauma & Reconstructive | Microsurgery | Europe | porcine |
| Clin Transplant. 2013 Jul-Aug;27 Suppl 25:6-15. doi: 10.1111/ctr.12155.  Do we need animal hands-on courses for transplantation surgery?  Golriz M, Hafezi M, Garoussi C, Fard N, Arvin J, Fonouni H, Nickkholgh A, Kulu Y, Frongia G, Schemmer P, Mehrabi A. | 2013 | Abdominal | Open surgery | Europe | porcine |
| J Surg Res. 2018 Feb;222:132-138. doi: 10.1016/j.jss.2017.09.042. Epub 2017 Nov 4.  Establishment of laparoscopic live donor nephrectomy in a porcine model: techniques and outcomes in 44 pigs. Newman ME, Musk GC, He B. | 2018 | Abdominal | Laparoscopic surgery | Australia | porcine |
| Ann Thorac Surg. 2018 Feb;105(2):637-643. doi: 10.1016/j.athoracsur.2017.10.011. Epub 2017 Dec 21.  Simulation and Deliberate Practice in a Porcine Model for Congenital Heart Surgery Training.  Mavroudis CD, Mavroudis C, Jacobs JP, DeCampli WM, Tweddell JS | 2018 | Cardiothoracic | Open surgery | North America | porcine |
| Ann Chir Plast Esthet. 2018 Apr;63(2):113-116. doi: 10.1016/j.anplas.2017.11.010. Epub 2017 Dec 26.  A porcine model for robotic training harvest of the rectus abdominis muscle.  Louis V, Chih-Sheng L, Chevallier D, Selber JC, Xavier F, Liverneaux PA | 2018 | Trauma & Reconstructive | Robotic surgery | europe | porcine |
| J Robot Surg. 2018 Dec;12(4):693-698. doi: 10.1007/s11701-018-0806-5. Epub 2018 Mar 31.  Robotic kidney autotransplantation in a porcine model: a procedure-specific training platform for the simulation of robotic intracorporeal vascular anastomosis.  Tiong HY, Goh BYS, Chiong E, Tan LGL, Vathsala A | 2018 | Trauma & Reconstructive | Robotic surgery | Asia | porcine |
| González-García JA, Chiesa-Estomba CM, Álvarez L, Altuna X, García-Iza L, Thomas I, Sistiaga JA, Larruscain E. Porcine experimental model for perforator flap raising in reconstructive microsurgery. | 2018 | Trauma & Reconstructive | Microsurgery | europe | porcine |
| J Robot Surg. 2019 Apr;13(2):289-292. doi: 10.1007/s11701-018-0852-z. Epub 2018 Jul 16.  Simulated management of urinary tract injury during robotic pelvic surgery utilizing the porcine model.  Hoffman MS, Spiess PE | 2018 | Urology | Robotic surgery | North America | porcine |
| Tayebi Meybodi A, Aklinski J, Gandhi S, Preul MC, Lawton MT.  Side-to-Side Anastomosis Training Model Using Rat Common Carotid Arteries. Oper Neurosurg (Hagerstown). 2019 Mar 1;16(3):345-350. doi: 10.1093/ons/opy157. | 2019 | Neurosurgery | Microsurgery | North America | murine (rat) |
| Leuzzi S, Maruccia M, Elia R, Annoscia P, Vestita M, Nacchiero E, Giudice G. Lymphatic-venous anastomosis in a rat model: A novel exercise for microsurgical training.  J Surg Oncol. 2018 Nov;118(6):936-940. doi: 10.1002/jso.25234. Epub 2018 Sep 27. | 2018 | Trauma & Reconstructive | Microsurgery | Europe | murine (rat) |
| J Vis Exp. 2018 Sep 29;(139). doi: 10.3791/58104.  Porcine As a Training Module for Head and Neck Microvascular Reconstruction.  Alessa MA, Kwak SH, Lee YW, Kang ML, Sung HJ, Ahn SH, Choi EC, Kim WS. | 2018 | Trauma & Reconstructive | Microsurgery | Asia | porcine |
| Ear Nose Throat J. . doi: 10.1177/0145561319840835.  Training Residents to Perform Tracheotomy Using a Live Swine Model.  Cheng PC, Cho TY, Hsu WL, Lo WC, Wang CT, Cheng PW, Liao LJ. | 2017 | Cardiothoracic | Open surgery | Europe | porcine |
| Eur J Vasc Endovasc Surg. 2018 Sep;56(3):373-380. doi: 10.1016/j.ejvs.2018.05.024. Epub 2018 Jul 11.  Experimental Evaluation of Endovascular Fenestration Scissors in an Ovine Model of Aortic Dissection.  El Batti S, Ben Abdallah I, Dufetelle E, Julia P, Menasche P, Alsac JM. | 2018 | Trauma & Reconstructive | Microsurgery | Europe | porcine |
| Int J Surg. 2018 Dec;60:245-251. doi: 10.1016/j.ijsu.2018.11.017. Epub 2018 Nov 24.  Critical steps for initiating an animal uterine transplantation model in sheep: Experience from a case series. Favre-Inhofer A, Carbonnel M, Revaux A, Sandra O, Mougenot V, Bosc R, Gélin V, Rafii A, Hersant B, Vialard F, Chavatte-Palmer P, Richard C, Ayoubi JM | 2018 | Abdominal | Open surgery | europe | porcine |
| Neurosurg Focus. 2019 Feb 1;46(2):E17. doi: 10.3171/2018.11.FOCUS18533.  Novel rodent model for simulation of sylvian fissure dissection and cerebrovascular bypass under subarachnoid hemorrhage conditions: technical note and timing study.  Perry A, Graffeo CS, Carlstrom LP, Anding WJ, Link MJ, Rangel-Castilla L | 2019 | neurosurgery | Microsurgery | North America | murine (rat) |
| Int Urogynecol J. 2017 Oct;28(10):1573-1577. doi: 10.1007/s00192-017-3313-8. Epub 2017 Mar 20.  Feasibility and benefits of the ewe as a model for vaginal surgery training.  Kerbage Y, Giraudet G, Rubod C, Garabedian C, Rivaux G, Cosson M. | 2017 | Urology | Open surgery | Europe | ovine |
| Transplant Proc. 2016 Nov;48(9):3053-3058. doi: 10.1016/j.transproceed.2016.07.032.  Establishing the Number of Procedures for Optimal Renal Transplantation Training With the Use of a Canine Model. Ayala-Garcia MA, Soel-Encalada JM, Rios Zambudio A, Rodea-Montero ER, Gonzalez-Yebra B. | 2016 | Abdominal | Open surgery | North America | lapine (rabbit) |
| World J Gastrointest Surg. 2016 Nov 27;8(11):735-743.  Impact of laparoscopic surgery training laboratory on surgeon’s performance.  Torricelli FC, Barbosa JA, Marchini GS. | 2016 | Abdominal | Laparoscopic surgery | South America | porcine |
| J Reconstr Microsurg. 2017 Jul;33(6):426-434. doi: 10.1055/s-0037-1599101. Epub 2017 Mar 10.  A New Supermicrosurgery Training Model of Saphenous Artery and Great Saphenous Vein Anastomosis for Development of Advanced Microsurgical Skills.  Bas CE, Cwykiel J, Siemionow M. | 2017 | Trauma & Reconstructive | Microsurgery | North America | murine (rat) |
| Int Urogynecol J. 2017 Oct;28(10):1595-1597. doi: 10.1007/s00192-017-3292-9. Epub 2017 Mar 14.  Development of an ovine model for training in vaginal surgery for pelvic organ prolapse.  Mansoor A, Curinier S, Campagne-Loiseau S, Platteeuw L, Jacquetin B, Rabischong B. | 2017 | Abdominal | Open surgery | Europe | ovine |
| Ann Plast Surg. 2017 Sep;79(3):298-303. doi: 10.1097/SAP.0000000000001133.  Mastering Lymphatic Microsurgery: A New Training Model in Living Tissue.  Campisi CC, Jiga LP, Ryan M, di Summa PG, Campisi C, Ionac M. | 2017 | Trauma & Reconstructive | Microsurgery | Europe | porcine |
| J Surg Educ. 2018 Jan - Feb;75(1):195-199. doi: 10.1016/j.jsurg.2017.06.029. Epub 2017 Jul 12.  Surgical Training Improves Performance in Minimally Invasive Left Ventricular Assist Device Implantation Without Cardiopulmonary Bypass.  Zhang LF, Feng HB, Yu ZG, Jing S, Wan F. | 2018 | Cardiothoracic | Microsurgery | Asia | porcine |
| Surg Innov. 2017 Oct;24(5):533-535. doi: 10.1177/1553350617723533. Epub 2017 Aug 2.  Surgical Training With Live Animal Models for Laparoscopic Gastrectomy.  Ueda Y, Shiraishi N, Hirashita T, Etoh T, Inomata M, Kitano S. | 2017 | Abdominal | Laparoscopic surgery | Asia | porcine |
| Asian J Endosc Surg. 2018 May;11(2):151-154. doi: 10.1111/ases.12433. Epub 2017 Oct 17.  Proper training in laparoscopic hernia repair is necessary to minimize the rising recurrence rate in Japan.  Matsumoto S, Hayakawa T, Kawarada Y, Uchida K, Eguchi T, Wada H, Ueno N, Idani H, Nakano K, Oomomo Y. | 2018 | Abdominal | Laparoscopic surgery | Asia | porcine |
| J Vis Surg. 2017 May 25;3:72. doi: 10.21037/jovs.2017.03.24. eCollection 2017.  Swine model for training surgeons in minimally invasive anatomic lung segmentectomy.  Oizumi H, Kato H, Endoh M, Suzuki J, Watarai H, Hamada A, Suzuki K, Nakahashi K, Sadahiro M. | 2017 | Cardiothoracic | Endoscopic surgery | Asia | porcine |
| Surg Laparosc Endosc Percutan Tech. 2018 Feb;28(1):e24-e29. doi: 10.1097/SLE.0000000000000492.  Percutaneous Image-guided Surgery Training: Model IHU-DAICIM.  Gimenez ME, Davrieux CF, Serra E, Palermo M, Houghton EJ, Acquafresca P, Dallemagne B, Kwak JM, Gonzalez CA, Marescaux . | 2018 | Abdominal | Endoscopic surgery | South America | porcine |
| Surg Innov. 2019 Dec;26(6):738-743. doi: 10.1177/1553350619881068. Epub 2019 Oct 11. Development and Preliminary Validation of a Rabbit Model of Duodenal Atresia for Training in Pediatric Surgical Skills. Ordorica-Flores R, Orpinel-Armendariz E, Rodríguez-Reyna R, Pérez-Escamirosa F, Castro-Luna R, Minor-Martínez A, Nieto-Zermeño J. | 2019 | abdominal | Laparoscopic surgery | South America | lapine (rabbit) |
| J Reconstr Microsurg. 2019 Sep;35(7):499-504. doi: 10.1055/s-0039-1679957. Epub 2019 Mar 5. A Novel Rat Model for Comprehensive Microvascular Training of End-to-End, End-to-Side, and Side-to-Side Anastomoses. Yin X, Ye G, Lu J, Wang L, Qi P, Wang H, Wang J, Hu S, Yang X, Chen K, Wang D. | 2019 | Trauma & Reconstructive | Microsurgery | Asia | murine (rat) |
| Innovations (Phila). 2019 Feb;14(1):37-42. doi: 10.1177/1556984519828016. Epub 2019 Feb 15. Optimizing Surgical Skills in Cardiac Surgery Residents with Cardiac Transplant in the High-Fidelity Porcine Model. Spooner AJ, Faulkner CM, Novick RJ, Kent WDT. | 2019 | Cardiothoracic | Microsurgery | North America | porcine |
| Ear Nose Throat J. 2019 Aug;98(7):E87-E91. doi: 10.1177/0145561319840835. Epub 2019 Apr 11. Training Residents to Perform Tracheotomy Using a Live Swine Model. Cheng PC, Cho TY, Hsu WL, Lo WC, Wang CT, Cheng PW, Liao LJ. | 2019 | Trauma & Reconstructive | Open surgery | Asia | porcine |
| Int Urogynecol J. 2019 Aug;30(8):1371-1375. doi: 10.1007/s00192-019-03936-7. Epub 2019 May 4. A live porcine model for robotic sacrocolpopexy training. | 2019 | Urology | Robotic surgery | North America | porcine |
| Laryngoscope. 2019 Sep 30. doi: 10.1002/lary.28309. [Epub ahead of print] Live porcine model for surgical training in tracheostomy and open-airway surgery. | 2019 | Trauma & Reconstructive | Endoscopic surgery | North America | porcine |
| J Invest Surg. 2019 Oct 6:1-8. doi: 10.1080/08941939.2019.1663376. [Epub ahead of print] Establishment of a Canine Training Model for Digestive Tract Reconstruction after Pancreaticoduodenectomy. | 2019 | Abdominal | Open surgery | Asia | misc |
| J Robot Surg. 2019 Nov 18. doi: 10.1007/s11701-019-01036-8. [Epub ahead of print] Simulated management of inferior vena cava injury during robotic paraaortic lymphadenectomy utilizing the porcine model. | 2019 | Abdominal | Robotic surgery | North America | porcine |
| Injury. 2020 Feb 11. pii: S0020-1383(20)30080-2. doi: 10.1016/j.injury.2020.02.006. [Epub ahead of print] A sequence of flaps and dissection exercises in the living model to improve the learning curve for perforator flap surgery. | 2020 | Trauma & Reconstructive | Microsurgery | Europe | porcine |
| J Hand Surg Am. 2020 Mar 31. pii: S0363-5023(20)30077-0. doi: 10.1016/j.jhsa.2020.02.001. [Epub ahead of print] The Turkey Digit: A New Training Model for Digit Replantation. | 2020 | Trauma & Reconstructive | Microsurgery | North America | misc |
| Surg Innov. 2019 Dec;26(6):738-743. doi: 10.1177/1553350619881068. Epub 2019 Oct 11. Development and Preliminary Validation of a Rabbit Model of Duodenal Atresia for Training in Pediatric Surgical Skills. Ordorica-Flores R, Orpinel-Armendariz E, Rodríguez-Reyna R, Pérez-Escamirosa F, Castro-Luna R, Minor-Martínez A, Nieto-Zermeño. | 2019 | abdominal | Laparoscopic surgery | South America | lapine (rabbit) |

**S2: Excluded studies**

We excluded a total of 51 studies after review of the full-text articles. The reasons for exclusion from further analyses were:

- Review paper
- Ex-vivo model of surgical training
- No animal model described
- No details on the model used
- Perspective or Opinion article
- Similar model published by same authors
- No training models described
- Veterinary training

| Dini, G. M., et al. (2012). "Training rhinoseptoplasty, sinusectomy, and turbinectomy in an animal model." Plast Reconstr Surg 130(1): 224e-226e. |
| --- |
| Fiorelli, A., et al. (2015). "A home-made animal model in comparison with a standard manikin for teaching percutaneous dilatational tracheostomy." Interact Cardiovasc Thorac Surg 20(2): 248-253. |
| Izawa, Y., et al. (2016). "Ex-vivo and live animal models are equally effective training for the management of a penetrating cardiac injury." World J Emerg Surg 11(1): 45. |
| Maluf Junior, I., et al. (2014). "An alternative experimental model for training in microsurgery." Rev Col Bras Cir 41(1): 72-74. |
| Nasser Kotby, M., et al. (2012). "Animal model for training and improvement of the surgical skills in endolaryngeal microsurgery." J Voice 26(3): 351-357. |
| Ni, D., et al. (2014). "Laparoendoscopic single-site urethrovesical anastomosis training in an economical porcine model." Urol Int 92(1): 89-94. |
| Padhye, V., et al. (2015). "Coping with catastrophe: the value of endoscopic vascular injury training." Int Forum Allergy Rhinol 5(3): 247-252. |
| Pioche, M., et al. (2015). "New isolated bovine colon model dedicated to colonic ESD hands-on training: development and first evaluation." Surg Endosc 29(11): 3209-3215. |
| Seaman, D. L., et al. (2007). "An animal training model for endoscopic treatment of Zenker's diverticulum." Gastrointest Endosc 65(7): 1050-1053. |
| Shalhav, A. L., et al. (2002). "Training postgraduate urologists in laparoscopic surgery: the current challenge." J Urol 167(5): 2135-2137. |
| Shetty, S., et al. (2014). "Perceptions, training experiences, and preferences of surgical residents toward laparoscopic simulation training: a resident survey." J Surg Educ 71(5): 727-733. |
| Teh, S. H., et al. (2007). "A suitable animal model for laparoscopic hepatic resection training." Surg Endosc 21(10): 1738-1744. |
| Zenati, M. A., et al. (2002). "A new live animal training model for off-pump coronary bypass surgery." Heart Surg Forum 5(2): 150-151. |
| Mallmann LB^1^, Piltcher OB^2^, Isolan GR^3^.2016 The Lamb's Head as a Model for Surgical Skills Development in Endonasal Surgery.J Neurol Surg B Skull Base. 2016 Dec; |
| Cifuentes IJ1, Yañez RA1, Salisbury MC1, Rodriguez JR1, Varas JE2, Dagnino BL1. 2016 A Novel Perforator Flap Training Model Using a Chicken Leg.J Hand Microsurg. 2016 Apr;8 |
| Chark D^1^, Oliaei S, Manuel C, Wong BJ.Porcine cartilage model for simulation of nasal tip aesthetics and mechanics.Aesthet Surg J. 2011 Jul;31(5):501-5. doi: 10.1177/1090820X11411581. |
| Achar RA^1^, Lozano PA, Achar BN, Pereira Filho GV, Achar E.Experimental model for learning in vascular surgery and microsurgery: esophagus and trachea of chicken.Acta Cir Bras. 2011 Apr;26(2):101-6. |
| Baek RM^1^, Eun SC, Heo CY, Chang H.Experimental facial transplantation surgery.J Craniofac Surg. 2010 May;21(3):648-51. doi: 10.1097/SCS.0b013e3181d84010. |
| Sotelo RJ^1^, Astigueta JC, Carmona OJ, De Andrade RJ, Moreira OE.Chicken gizzard: a new training model for laparoscopic urethrovesical anastomosis.Actas Urol Esp. 2009 Nov;33(10):1083-7. |
| Vázquez-Sequeiros E1, de Miquel DB, Olcina JR, Martín JA, García M, Lucas DJ, Garrido E, González C, Blanco AP, Arnau MR, Buenadicha A, Vicente VM, de Argila CM, Milicua JM.Training model for teaching endoscopic submucosal dissection of gastric tumors.Rev Esp Enferm Dig. 2009 Aug;101(8):546-52. |
| Estaca E1, Cabezas J, Usón J, Sánchez-Margallo F, Morell E, Latorre R.Maxillary sinus-floor elevation: an animal model.Clin Oral Implants Res. 2008 Oct;19(10):1044-8. doi: 10.1111/j.1600-0501.2008.01557.x. |
| Phillips AB1, Green J, Bergdall V, Yu J, Monreal G, Gerhardt M, Cheatham JP, Galantowicz M, Holzer RJ.Teaching the "hybrid approach": a novel swine model of muscular ventricular septal defect.Pediatr Cardiol. 2009 Feb;30(2):114-8. doi: 10.1007/s00246-008-9297-x. Epub 2008 Aug 19. |
| Chen VK1, Marks JM, Wong RC, McGee MF, Faulx AL, Isenberg GA, Schomisc SJ, Deng CX, Ponsky JL, Chak A.Creation of an effective and reproducible nonsurvival porcine model that simulates actively bleeding peptic ulcers.Gastrointest Endosc. 2008 Sep;68(3):548-53. doi: 10.1016/j.gie.2008.03.1087. Epub 2008 Jul 11. |
| Postgate, A., et al. (2008). "The impact of experience on polyp detection and sizing accuracy at capsule endoscopy: implications for training from an animal model study." Endoscopy 40(6): 496-501. |
| Ioannou I, Kazmierczak E, Stern L. Comparison of oral surgery task performance in a virtual reality surgical simulator and an animal model using objective measures.Conf Proc IEEE Eng Med Biol Soc. 2015;2015:5114-7. doi: 10.1109/EMBC.2015.7319542. |
| Ioannou I, Kazmierczak E, Stern L.Comparison of oral surgery task performance in a virtual reality surgical simulator and an animal model using objective measures.Conf Proc IEEE Eng Med Biol Soc. 2015;2015:5114-7. doi: 10.1109/EMBC.2015.7319542. |
| Virtual training vs Animal model; Animal Model IMMER überlegen |
| Animal model beschrieben dass für Training entwickelt wurde |
| Rizvi RM1. Assessment of experimental animal model for training obstetric anal sphincter injury techniques.J Pak Med Assoc. 2013 Jan;63(1):103-5. |
| Martinek J1, Suchanek S, Stefanova M, Rotnaglova B, Zavada F, Strosova A, Zavoral M.Training on an ex vivo animal model improves endoscopic skills: a randomized, single-blind study.Gastrointest Endosc. 2011 Aug;74(2):367-73. doi: 10.1016/j.gie.2011.04.042. |
| Cordero A^1^, del mar Medina M, Alonso A, Labatut T.Stapedectomy in sheep: an animal model for surgical trainingOtol Neurotol. 2011 Jul;32(5):742-7. doi: 10.1097/MAO.0b013e31821ddbc2. |
| Al-Bustani, S. and E. G. Halvorson (2016). "Status of Microsurgical Simulation Training in Plastic Surgery: A Survey of United States Program Directors." Ann Plast Surg 76(6): 713-716. |
| Di Cataldo, A. and G. Li Destri (2007). "Do we still need experimental surgery and research?" Microsurgery 27(4): 346-347. |
| Ganpule, A., et al. (2015). "Chicken and porcine models for training in laparoscopy and robotics." Curr Opin Urol 25(2): 158-162. |
| Khan, R., et al. (2015). "Simulation-based training for prostate surgery." BJU Int 116(4): 665-674. |
| Tsuji, Y., et al. (2014). "Desirable training of endoscopic submucosal dissection: further spread worldwide." Ann Transl Med 2(3): 27. |
| Padhye, V., et al. (2015). "Coping with catastrophe: the value of endoscopic vascular injury training." Int Forum Allergy Rhinol 5(3): 247-252. |
| Bauer F et al (2015). „Special training in maxillofacial surgery for medical students--economic burden or investment in the future?” Br J Oral Maxillofac Surg. 2015 Dec;53(10):1012-4. doi: 10.1016/j.bjoms.2015.10.004. Epub 2015 Oct 28. |
| Loh C et al. Animal models in plastic and reconstructive surgery simulation-a review. J Surg Res. 2018 Jan;221:232-245. doi: 10.1016/j.jss.2017.08.052. Epub 2017 Sep 28. |
| [The animal models using live pigs in the application and development of endoscopic submucosal dissection training]. Li J, Zhong YS, Zhou PH, Chen T, Yao LQ.  Zhonghua Wei Chang Wai Ke Za Zhi. 2019 Jul 25;22(7):697-700. doi: 10.3760/cma.j.issn.1671-0274.2019.07.019. Chinese. |
| Role of live animals in the training of microvascular surgery: a systematic review. Brown JS, Rapaport BHJ. Br J Oral Maxillofac Surg. 2019 Sep;57(7):616-619. doi: 10.1016/j.bjoms.2019.06.003. Epub 2019 Jun 22. |
| Optimal refinement of residents' surgical skills by training on induced goat's eye corneoscleral perforation. Pujari A, Sharma N, Chaniyara MH, Urkude J, Singh R, Yadav S, Mukhija R, Asif MI, Sidhu N. Indian J Ophthalmol. 2019 Apr;67(4):547-548. doi: 10.4103/ijo.IJO_1474_18. |
| J Vis Exp. 2020 Mar 18;(157). doi: 10.3791/60407. Learning Modern Laryngeal Surgery in a Dissection Laboratory. Crosetti E1, Fantini M1, Lancini D2, Manca A1, Succo G3. |
| World J Urol. 2019 Sep;37(9):1879-1887. doi: 10.1007/s00345-018-2602-2. Epub 2018 Dec 17. A newly developed porcine training model for transurethral piecemeal and en bloc resection of bladder tumour. Teoh JY1, Cho CL2, Wei Y3, Isotani S4, Tiong HY5, Ong TA6, Kijvikai K7, Chu PS8, Chan ES9, Ng CF10; Asian Urological Surgery Training & Education Group. |
| World Neurosurg. 2019 Sep;129:55-61. doi: 10.1016/j.wneu.2019.05.199. Epub 2019 May 29. Experimental Model for Interlaminar Endoscopic Spine Procedures.  Amato MCM1, Aprile BC2, de Oliveira CA2, Carneiro VM3, de Oliveira RS3. |
| Obstet Gynecol. 2019 Jul;134(1):163-168. doi: 10.1097/AOG.0000000000003333. A Novel Porcine Stomach Tissue Model for Laparoscopic Colpotomy Simulation.  Cho M1, Ulrich A, Lam C, Lerner V. |
| Neurosurg Focus. 2019 Jul 1;47(1):E20. doi: 10.3171/2019.4.FOCUS19219. In vivo cerebral aneurysm models.  Thompson JW1,2, Elwardany O1,2, McCarthy DJ1,2, Sheinberg DL1,2, Alvarez CM1,2, Nada A1,2, Snelling BM1,2,3, Chen SH1,2, Sur S1,2, Starke RM1,4,2. |

**S3: Search strategy**

Database: PubMed, Google Scholar, Web of Science core collection and Scopus. Date of the last search: March 31^st^, 2020

1. Surgery training
2. Surgical training
3. Animal model "[MeSH Terms]
4. Animal training
5. training model
6. animal*
7. in-vivo model
8. #1 -2 OR
9. #3-7 OR
10. NOT ex-vivo
11. NOT exvivo
12. NOT review
13. NOT literature analysis
14. NOT perspective
15. #12-14 OR
